# Factorial Structure and Cross-Cultural Invariance of the Parenting Stress Index-Short Form in Hong Kong and Thailand

**DOI:** 10.3389/fpsyg.2021.661972

**Published:** 2021-05-13

**Authors:** Xiaozi Gao, Kerry Lee

**Affiliations:** Centre for Educational and Developmental Sciences, The Education University of Hong Kong, Ting Kok, Hong Kong

**Keywords:** parenting stress, test adaptation, measurement invariance, convergent and discriminant validity, cross-culture

## Abstract

With increasing attention on the role of parenting stress on family functioning and children’s development, one area that has been neglected is how such relations differ across cultures. Although sometimes viewed as homogeneous, Asian countries often have markedly different belief systems. Cross-cultural studies require instruments that have been validated in different socio-cultural contexts. The widely used parenting stress index-short form (PSI-SF) has been used in several locations. However, results regarding its factorial structure have been mixed. Furthermore, there are only a few cross-cultural comparison studies. This study examined the factorial structure of an abridged version of the PSI-SF with data from Hong Kong (*N* = 258) and Thailand (*N* = 190). The results from confirmatory factor analyses indicated that, in both cultures, a three-factorial structure provides the best model fit. Furthermore, we found evidence for partial metric invariance, suggesting that the test scores can be compared directly. Tests for convergent and discriminant validity revealed that the three factors were correlated with parent general distress, authoritative, authoritarian, and permissive parenting behaviors, in both cultures. These findings suggest that the abridged PSI-SF can provide a meaningful comparison of parenting stress between Hong Kong and Thailand.

## Introduction

Although raising a child can provide parents with joy and a sense of meaning in life, daily interaction with children can sometimes be challenging and perceived as stressful by parents (Nomaguchi and Milkie, 2003). Parenting stress, typically defined as individuals’ perception of the difficulties and feelings in not being able to meet the demands of being a parent (Abidin, 1992; Crnic and Low, 2002; Deater-Deckard, 2008), has received much attention during the past decades. The available evidence suggests that excessive parenting stress reduces the use of positive parenting behaviors, such as warmth and responsiveness (Fonseca et al., 2020; Ward and Lee, 2020; de Maat et al., 2021), and is associated with higher levels of negative parenting behaviors, for instance, over-reactivity, and permissiveness (Fonseca et al., 2020; Mak et al., 2020; de Maat et al., 2021), regardless of whether children have developmental disabilities (Ueda et al., 2020). There is also evidence to suggest that such stress-related effects are enduring in nature and affect children’s social–emotional, behavioral, and cognitive development (Bosquet Enlow et al., 2019; Cherry et al., 2019; Mak et al., 2020; Ward and Lee, 2020; Kochanova et al., 2021). Although the majority of studies about parenting stress were conducted in western and Anglo-European countries (Touchèque et al., 2016; Rivas et al., 2020; de Maat et al., 2021), an increasing number of studies are conducted in Asia (Ilias et al., 2018; Lau and Power, 2020; Mak et al., 2020).

According to the bioecological perspective of parenting (Bronfenbrenner and Morris, 2007), the broad cultural and societal contexts instil values, beliefs, or expectations in parents, which shape how people perceive, recognize, and cope with stress (Chun et al., 2006). Although there are studies comparing the stress levels of parents from Asia and the United States (e.g., Chung et al., 2013; Nomaguchi and House, 2013), few have compared parenting stress among Asian countries. Asian culture is by no means homogeneous (Keats, 2000). In an area related to but distinct from parenting stress, Ng (2002) argued that people from the Far East, especially those from societies with higher economic growth and Confucian cultures (e.g., Singapore, Hong Kong, or Japan), were less happy compared to those from Southeast Asian societies (e.g., the Philippines or Thailand). Ng (2002) argued that Confucianism’s emphasis on achievement may render such societies more competitive, with a tendency for people to try to surpass others, and not being content with their current achievement—all of which are likely to induce stress and undermine happiness. It seems likely that elements of this competitiveness will extend to parenting practices and produce a parenting stress gap between Far Eastern and Southeast Asian societies.

Indeed evidence of such competitiveness in parenting practices abound. In Hong Kong, local parents typically make every effort to ensure that their children “win at the start.” Even kindergarten children are often sent to tutoring schools and are enrolled in extracurricular activities to enhance the chances of future success (Hong Kong Institute of Asia-Pacific Studies, 2016). In comparison, Thais tend to be more heavily influenced by Buddhist beliefs that emphasize the virtue of contentedness and the undesirable consequences of competition (Ng, 2002). Furthermore, in Buddhism, stress is seen as an inevitable part of human existence which is built into the physical body in a form of dukkha (Tyson and Pongruengphant, 2007). Given the pervasiveness of Buddhist beliefs in Thai society, as compared to Hong Kong, it is likely that Thais both experience less parenting stress and have a higher tolerance to stress than their Hong Kong counterparts. To what extent such traditional cultural beliefs are over-ridden by the competitiveness of urban living in modern Thai societies is unclear. To examine differences in parenting stress, it is vital that we use an instrument that provides valid and reliable measures for both societies. In this study, we examined the suitability of an abridged version of the Parenting Stress Index (PSI; Abidin, 1995).

### Measurement of Parenting Stress

The original version of the widely used PSI consists of 101 items that assess stress associated with two dimensions: parenthood and child-rearing (Abidin, 1983). Subsequently, Abidin (1995) developed an abbreviated version parenting stress index-short form (PSI-SF) that consisted of 36 items measuring three dimensions: parental distress (PD; 12 items), parent–child dysfunctional interaction (PCDI; 12 items), and difficult child (DC; 12 items). To further reduce subject burden, Yeh et al. (2001) developed a 15-item abridged version and found that it provided comparable reliability and validity as the PSI-SF.

The three-factorial structure has been found in studies conducted in different cultures, including Spanish (Rivas et al., 2020), French (Touchèque et al., 2016), Jordanian (Dardas and Ahmad, 2014), Chilean (Aracena et al., 2016), and mainland Chinese (Luo et al., 2019). However, support for alternative structures had also been found. Haskett et al. (2006), for example, worked with a sample of 185 African American and Caucasian parents and found that the instrument was better defined by a two-factor structure consisting of PD and a second child-rearing stress factor consisting of the PCDI and DC subscales. Their findings support Abidin’s (1983) earlier theory that specified two dimensions of parenting stress. Pérez-Padilla et al. (2015) found a similar two-factor structure among a sample of at-risk and not-at-risk Spanish mothers. In a sample of 192 African American and Caucasian low-income mothers, Reitman et al. (2002) found that the most parsimonious single-factor structure had essentially the same fit as the two- and three-factor structure. However, in examining the discriminant validity of the three dimensions, they found each dimension to be related to other relevant constructs in different ways. For this reason, they recommended the three-factor model for clinical purposes. Aracena et al. (2016) argued that a two-factor solution is favored when samples include both fathers and mothers, with a three-factor model favored when only mothers are involved. However, two recent studies that included both parents supported the three-factor structure (Touchèque et al., 2016; Luo et al., 2019). In addition to studies that stayed close to the dimensions postulated by Abidin (1983, 1995), others found that PD and PCDI could be decomposed into five narrower factors (Whiteside-Mansell et al., 2007; McKelvey et al., 2009). From the brief review above, it can be seen that some doubts remain as to whether a one-, two-, or three-factor solution best describes the PSI-SF.

### The Current Study

The present study has two aims. First, we conducted confirmatory factor analyses (CFA) to examine whether the factorial structure of the abridged PSI-SF differed across Hong Kong and Thailand. Three-factorial configurations were examined: (a) a one-factor model with all 15 items loading onto one general parenting stress factor (Reitman et al., 2002), (b) a two-factor model that is comprized of a PD and a child-rearing stress (PCDI + DC) factor (Haskett et al., 2006), and (c) a three-factor model with PD, PCDI, and DC (Aracena et al., 2016). A three-factor structure has been found among mainland Chinese (Luo et al., 2019). Although there are some cultural differences between the mainland and Hong Kong, both are rooted in Confucian and traditional Chinese culture. For this reason, we hypothesized that a similar three-factorial structure would be found among Hong Kong parents. Although Srikosai et al. (2020) also found that a three-factorial structure fitted the data from Thai parents, we could not find prior studies that have examined more detailed aspects of measurement invariance. This issue was examined using a multigroup CFA to test whether the S-PSI-SF differed across the two locations. If measurement invariance is found and scores can be meaningfully compared, we expected the parenting stress of Thai parents to be lower than that of Hong Kong parents.

We also examined differences in the convergent and discriminant validity of the S-PSI-SF by examining the relations between each subscale and several related constructs, including parents’ general distress and parenting style. We expected all three factors to demonstrate moderate to strong positive correlations with parents’ general distress because both instruments measured the parent’s mental health-related constructs. However, because PCDI, PD, and DC measured stress derived from different causes (PCDI and DC measure parenting stress resulting from interacting with or regulating the children, while PD assesses stress derived from the constraints on life resulting from parenthood; Abidin, 1995), we anticipated differences in how the three subscales correlate with other measures. Specifically, based on previous findings which found that PD explained variance in parents’ depression even after controlling for PCDI and DC (Luo et al., 2019), we expected PD to be more strongly related to general distress than do PCDI and DC in both societies.

We also expected all three subscales to be positively associated with authoritarian and permissive parenting and negatively related to authoritative parenting. Previous studies showed that parenting stress hampers the quality of parenting behaviors (Haskett et al., 2006; Luo et al., 2019). Previous studies also suggested that PD was not related to parenting style when PCDI and DC were included as explanatory models (Haskett et al., 2006). For this reason, we expected PCDI and DC to be more strongly associated with parenting than does PD in both locations. Specifically, PCDI and DC were expected to be more strongly and negatively associated with authoritative parenting and more strongly and positively associated with authoritarian and permissive parenting than PD.

## Materials and Methods

### Participants

The data was part of a larger international project investigating the relationship between family social economic status (SES) and children’s cognitive development. To attenuate differences that may result from a rural versus an urban setting, we focused our data collection effort in Bangkok, the urban administrative and commercial capital of Thailand. To achieve a broader coverage, we selected and recruited children and their parents from kindergartens located in lower, medium, and higher SES districts in Bangkok and Hong Kong. Because of differences in access, there were some differences in the number of kindergartens that we were able to recruit in the two locations. In Hong Kong, we recruited children from 13 kindergartens, with five located in the lower, four in the middle, and four in higher SES districts. In Bangkok, we recruited five kindergartens, with one from the lower, one from the middle, and three from the middle to higher SES districts. The full sample contained 258 Hong Kong parent–child dyads (95 girls, *M* = 69.72 months, SD = 4.75) and 190 Thai parent–child dyads (88 girls, *M* = 70.58 months, SD = 8.52, excluding 32 Thai parents who did not finish the questionnaire).

Most of the respondents are mothers in both Hong Kong (84.9% mothers, 14.7% fathers, and 0.4% grandparents) and Bangkok (73.5% mothers, 16.4% fathers, and 10% grandparents). Of the Hong Kong parents, 50.4% had university degrees, 21.7% had other post-secondary education, and 27.5% completed secondary education or below. Among the Thai parents, 31.3% had completed secondary education or below, 13.2% had completed other post-secondary education, and 32.1% had university or master’s degrees. Our Hong Kong parents’ median monthly household income was between HKD 50,001 and HKD 60,000. Although their median monthly household income is relatively higher than that indicated in the census data (HKD 35,000, Census and Statistics Department, 2019), our Hong Kong parents’ median household monthly income per capita is between HKD 10,000 and HKD 20,000, similar to that found in the census data (∼HKD10,900). Our Thai sample’s median monthly household income was between THB 18,001 and THB 30,000, relatively lower than the census data (THB 39,459; National Statistical Office, 2019). Their median monthly household income per capita is around THB 3,000 (census range is THB 10,000–15,000). Both values showed that the income of our Thai sample is lower than that found in the general population.

### Procedure

The study protocol was approved by the ethics committees of the respective universities in Hong Kong and Thailand. Information sheets and consent forms were distributed to parents of participating kindergartens by the teachers. These were followed up with a parent questionnaire that assessed family SES, parenting behavior, parenting stress, and mental health. The parents were informed that there were no incorrect answers for each question. The parents were provided, upon completion of the questionnaire, a supermarket coupon of HKD 100 in Hong Kong and THB 200 in Bangkok.

### Measures

We used the abridged PSI-SF to measure self-reported parenting stress (Yeh et al., 2001). The parents were asked to respond to 15 items on a five-point Likert scale from strongly disagree to strongly agree. A higher score represents a higher level of parenting stress.

The parents’ mental health was measured using the six-item version of Kessler’s psychological distress scale (Kessler et al., 2003). They were asked to indicate how often they experienced various distress (e.g., hopelessness or nervousness) in the past 30 days. The average scores of each scale were calculated, with a higher score representing a higher level of distress. Cronbach’s α was 0.874 for the Hong Kong sample and 0.941 for the Thai sample.

Parenting behaviors were measured using the 32-item Parenting Styles and Dimensions Questionnaire (PSDQ; Robinson et al., 2001). The PSDQ measured three styles of parenting: authoritative, authoritarian, and permissive. The parents indicated how often they engaged in various behaviors (e.g., “allow the child to give input into family rules” or “spanks when the child is disobedient”) using a five-point Likert scale. Mean scores were generated for each subscale, with a higher score indicating higher reliance on a parenting style. Cronbach’s α was 0.882 for authoritative parenting in Hong Kong and 0.878 in Thailand, 0.852 for authoritarian parenting in Hong Kong and 0.896 in Thailand, and 0.651 for permissive parenting in Hong Kong and 0.579 in Thailand.

### Analytical Plan

The analysis was conducted in two phases. First, we examined the factorial structure of the abridged PSI-SF (one-factor, two-factor, and three-factor) for Hong Kong and Bangkok separately. Visual inspection and Shapiro–Wilk test revealed that some of the items were highly skewed. Therefore, we used the robust weighted least squares estimator (WLSMV) in Mplus 8.5 (Muthén and Muthén, 2018). The WLSMV estimator is recommended when the data are not normally distributed (Brown, 2015). Model fit was evaluated using chi-square values (*p* > 0.05), comparative fit indices (CFI > 0.90), root mean square error of approximation (RMSEA < 0.08), and standardized root mean residual (SRMR < 0.08) (Hu and Bentler, 1999). Latent factor reliability was calculated using McDonald’s omega coefficient (ω) to evaluate internal consistency (McDonald, 1999). Although the Cronbach alpha coefficient (α) is a more popular measure of internal consistency, ω was used because it does not assume that the items have the same loadings and outperform α when error covariance exists (Yang and Green, 2011; Dunn et al., 2014).

Cross-cultural invariance was examined in the second phase. A series of multiple-group CFAs with increasing invariance restrictions (configural, metric, and scalar) was fitted to the combined dataset containing both the Hong Kong and Bangkok data. Cross-cultural measurement invariance is established if the model fit of the more restricted model is not significantly worse than the less restricted model. Given the sensitivity of the chi-square difference test to sample size, we focused on changes in CFI (>0.01) and RMSEA (>0.015) (Chen, 2007). If measurement invariance was established, mean structure invariance was tested by constraining latent factor means to be equal across groups.

## Results

### Factorial Structure

Table 1 presents the results of CFA or the one-, two-, and three-factor models. In both locations, the three-factor structure provided the best fit. Because the RMSEA for the three-factor model was relatively high, we examined the sources of misfit. The modification indices suggested that freeing the covariance between two items would decrease misfit. Because both items explicitly referred to children’s laughter and are conceptually related, freeing the covariance seems reasonable. Also suggested by the modification indices was a covariance between two items that explicitly referred to the parents’ perception of children’s emotionality. Freeing these two residual covariances improved the model fit for both Hong Kong and Thai samples. Most of the loadings onto their corresponding factors were high (>0.526; see Table 2). The only exception was one parental distress item in the Thai sample (*λ* = 0.151, *p* = 0.037).

**TABLE 1:** Model fit statistics of the three factorial structures for each culture.

**Model**	***χ*^2^**	**df**	**CFI**	**TLI**	**RMSEA**	**SRMR**	****Δ***χ*^2^**	****Δ** CFI**	****Δ** RMSEA**
**HK**									
°One-factor	949.310	90	0.759	0.719	0.194	0.128			
°Two-factor	399.713	89	0.913	0.897	0.117	0.080	135.727***	0.154	0.073
°Three-factor	326.572	87	0.933	0.919	0.104	0.067	42.227***	0.020	0.013
°Three-factor +2 MI	257.202	85	0.952	0.940	0.089	0.061	69.185***	0.019	0.015
**Thailand**									
°One-factor	640.269	90	0.824	0.795	0.179	0.099			
°Two-factor	410.039	89	0.897	0.879	0.138	0.077	70.17***	0.073	0.041
°Three-factor	331.145	87	0.922	0.906	0.122	0.066	48.565***	0.025	0.016
°Three-factor +2 MI	236.532	85	0.952	0.940	0.097	0.057	63.519***	0.030	0.025

^***^p < 0.001.

**TABLE 2:** Standardized factor loadings and reliability of subscales for each culture.

	**PD**			**PCDI**			**DC**	
**Item**	**HK**	**Thailand**	**Item**	**HK**	**Thailand**	**Item**	**HK**	**Thailand**
1	0.622***	0.151*	6	0.817***	0.823***	11	0.658***	0.710***
2	0.777***	0.526***	7	0.886***	0.885***	12	0.732***	0.725***
3	0.832***	0.796***	8	0.725***	0.758***	13	0.648***	0.626***
4	0.807***	0.831***	9	0.616***	0.835***	14	0.695***	0.652***
5	0.788***	0.765***	10	0.714***	0.765***	15	0.708***	0.898***
Reliability	0.715	0.847		0.870	0.885		0.862	0.778

*p < 0.05, ***p < 0.001.Reliability refers to McDonald’s omega (*ω*) coefficient.

### Cross-Cultural Invariance

The configural invariance model with two residual covariances (see M1 in Table 3) yielded a good model fit. After constraining all the factor loadings to be equal across groups, the metric invariance model showed an acceptable model fit (see M2 in Table 3). Using Chen’s (2007) cutoff, M2 was no worse than M1 (ΔCFI = 0.008 and ΔRMSEA = 0.003).

**TABLE 3:** Model fit statistics of the three factorial structures for each culture.

**Model**	***χ*^2^**	**Df**	**CFI**	**TLI**	**RMSEA**	**SRMR**
M1. Configural invariance	494.300	170	0.951	0.939	0.093	0.060
M2. Full metric invariance	555.373	182	0.943	0.935	0.096	0.068
M3. Full scalar invariance	800.318	239	0.915	0.925	0.103	0.072
M4. Partial^a^ scalar invariance	645.779	229	0.937	0.942	0.090	0.070
M5. Factor means invariance	605.032	232	0.943	0.949	0.085	0.070

*^*a*^Thresholds 3 and 4 of item 1, thresholds 2–4 of item 2, thresholds 1 and 2 of item 10, and thresholds 1–3 of item 11.*

The full scalar invariance model (M3 in Table 3), in which both factor loadings and intercepts were constrained to equality across groups, was worse than the metric model on only one of the two indices (ΔCFI = 0.028). The modification indices suggested that freeing the thresholds of four items will significantly improve model fit (M4). The fit of the resultant model was no worse than M2 (ΔCFI = 0.006, and ΔRMSEA = 0.002).

Given the finding of partial scalar invariance, we examined the factor mean difference following the suggestions of Vandenberg and Lance (2000). After constraining the factor means to be equal between groups (M5 in Table 3), the model fit did not deteriorate, indicating that the latent means were invariant across groups (ΔCFI = 0.006, ΔRMSEA = 0.005).

### Internal Consistency and Construct Validity

As shown in Table 2, reliability was high for all subscales in Hong Kong (>0.715) and Bangkok (0.778). In Hong Kong, all three subscales displayed moderate to large associations with the Kessler (0.372 < *r* < 0.424, see Table 4) and with the three parenting styles (−0.256 > *r* > −0.490). In Thailand, all three subscales were moderately related to the Kessler (0.199 < *r* < 0.252). In terms of parenting style, the results showed that PCDI was correlated moderately to strongly with the three parenting styles (−0.284 < *r* < 0.553). PD was only related to authoritarian parenting, and DC was related to authoritarian and permissive parenting styles.

**TABLE 4:** Correlations between subscales and related constructs.

	**Hong Kong**			**Thailand**		
**Subscales**	**PD**	**PCDI**	**DC**	**PD**	**PCDI**	**DC**
**PD**						
PCDI	0.425***			0.594**		
DC	0.499**	0.794**		0.574***	0.854***	
**Related constructs**						
Kessler	0.410***	0.424***	0.372***	0.244**	0.252**	0.199**
Authoritative	−0.256**	−0.490***	−0.411***	–0.124	−0.284***	–0.137
Authoritarian	0.305***	0.457***	0.415**	0.184*	0.553***	0.482***
Permissive	0.266***	0.342***	0.410***	0.102	0.400***	0.374***

*p < 0.05, **p < 0.01, ***p < 0.001.

Regarding discriminant validity, in Hong Kong, both PCDI and DC were significant explanatory variables of the Kessler, but DC became non-significant after PD was entered (see Table 5). PD predicted additional variance in parents’ general distress after controlling for the variance explained by PCDI and DC. In Thailand, PCDI explained the variance in parents’ general distress when PCDI and DC were entered together. PCDI was rendered non-significant when PD was entered. In the full regression model, PD was the only significant explanatory variable of the Kessler. This is partially consistent with our hypothesis that PD is a unique predictor of parents’ general distress.

**TABLE 5:** Hierarchical regressions: parenting stress subscales predicting general distress.

	**Hong Kong**		**Thailand**	
	***β***	***R*^2^**	***β***	***R*^2^**
**Step 1**				
PCDI	314***		0.211*	
DC	0.187**	0.203	0.064	0.066
**Step 2**				
PCDI	0.272***		0.178	
DC	0.103		0.019	
PD	0.282***	0.270	0.172*	0.091

*β*, standardized coefficient.*p < 0.05, **p < 0.01, ***p < 0.001.

In terms of parenting style, PD is a significant predictor of all three parenting styles in Hong Kong (see Table 6); however, the explanatory power of PD was rendered non-significant by PCDI and DC for authoritative and permissive parenting. Although PD remains significant for authoritarian parenting, its effect size was reduced. Both PCDI and DC explained the variance in authoritative and authoritarian parenting, but only DC was a significant explanatory variable of permissive parenting. Despite the strong correlation (*r* = 0.794) between PCDI and DC, DC but not PCDI was a significant explanatory variable for permissive parenting, indicating some distinctions between the two factors. In contrast, in Thailand, PD became non-significant for all parenting styles after PCDI and DC were included. The same as in Hong Kong, PCDI and DC correlated strongly and were significant explanatory variables for authoritarian parenting. However, in contrast to Hong Kong, only PCDI explained the variance in authoritative parenting, and both PCDI and DC explained the variance in permissive parenting.

**TABLE 6:** Hierarchical regressions: parenting stress subscales predicting parenting style.

	**Authoritative**				**Authoritarian**				**Permissive**			
	**Hong Kong**		**Thailand**		**Hong Kong**		**Thailand**		**Hong Kong**		**Thailand**	
	***β***	***R*^2^**	***β***	***R*^2^**	***β***	***R*^2^**	***β***	***R*^2^**	***B***	***R*^2^**	***β***	***R*^2^**
**Step 1**												
PD	−0.257***	0.066	–0.124	0.015	0.305***	0.093	0.184*	0.034	0.266***	0.071	0.102	0.010
**Step 2**												
PD	–0.073		–0.039		0.134*		–0.062		0.108		–0.091	
PCDI	−0.367***		−0.323**		0.309***		0.426***		0.137		0.291**	
DC	−0.169*	0.268	0.083	0.085	0.183**	0.259	0.235**	0.337	0.286***	0.193	0.224*	0.191

*β*, standardized coefficient.*p < 0.05, **p < 0.01, ***p < 0.001.

## Discussion

To advance the understanding of parenting stress in different societies, it is imperative to demonstrate whether an instrument is invariant between societies. The purpose of the study was to examine the factorial structure and the measurement invariance of the abridged PSI-SF between Hong Kong and Thailand and to test whether parenting stress differed. Consistent with previous findings (e.g., Touchèque et al., 2016), the results demonstrated that the three-factorial structure was supported in both cultures. The multigroup CFA further indicated that the three-factorial structure holds in both cultures. Additionally, partial scalar invariance was established, and factor mean invariance was supported. In both cultures, the S-PSI-SF demonstrated acceptable internal consistency and satisfactory convergent and discriminant validity in relating to parental general distress and parenting styles. Overall, the results suggested that the abridged PSI-SF could be used to measure and compare parenting stress in both Hong Kong and Bangkok.

Although Luo et al. (2019) argued that the original three-factor structure of S-PSI-SF was specific to clinical samples, our results suggest that it is also applicable to non-clinical samples in both sites, consistent with arguments that cultural or risk-related characteristics do not invalidate the use of the instrument (Lee et al., 2016). Surprisingly, latent mean invariance was supported. We did not find a “stress gap” between the two cultures. One possible reason is that, similar to Hong Kong, Bangkok’s urban parents are also placing more emphasis on their children’s early achievement. To make sure that their children attend good tutoring schools, parents have to work harder to pay for it, causing higher stress in them.

We found good internal consistency and evidence of convergent and discriminant validity for the three subscales in both sites. All three subscales demonstrated high model reliability. As hypothesized by and consistent with prior studies (e.g., Luo et al., 2019), a higher level of PD, PCDI, and DC is related to higher levels of general distress and higher identification with authoritarian and permissive parenting but a lower level of authoritative parenting in Hong Kong. Partially consistent with previous research in Thailand (e.g., Likhitweerawong et al., 2020), higher levels of PD, PCDI, and DC are all related to higher levels of general distress and authoritarian parenting. However, only PCDI was related to less authoritative parenting, and only PCDI and DC were related to higher permissive parenting in Thailand. These findings suggest that the correlation between parenting stress and parenting depends on stressors, that is, it matters whether the stressors arise from the child or parent–child interaction.

The hierarchical regression analysis suggests good discriminant validity. First, PD explained the variance in parents’ general distress even after controlling for PCDI and DC in both societies. These findings are consistent with previous research, which consistently found that PD was related to the parents’ mental health (Haskett et al., 2006; Luo et al., 2019). Second, our results are partially consistent with those of Luo et al. (2019) in terms of parenting behaviors. They show that PCDI and DC explained more variance in authoritative and authoritarian parenting than PD. Although Haskett e*t al*. (2006) suggested combining PCDI and DC as one factor, we found evidence of differentiation in both societies, despite of a different pattern. DC is the only explanatory variable of permissive parenting, independently of PD and PCDI in Hong Kong. PCDI and DC both explained the variance in permissive parenting in Thailand. PCDI and DC both explained the variance in authoritative parenting in Hong Kong, but only PCDI explained authoritative parenting in Thailand. A possible explanation for the difference between ours and the findings of Haskett et al. (2006) is that they used harsh discipline as a criterion variable. In contrast, we found a differentiation between PD and PCDI when we focused on permissiveness and authoritativeness. These results are consistent with the view that parenting stress is a multidimensional construct with the three dimensions, as suggested by Abidin (1995).

Several limitations need to be acknowledged. First, the present study only involved kindergarten children. Research has shown that parenting stress increases as children transit to adolescents (Putnick et al., 2010). Because the PSI-SF is developed for children up to 12 years old (Abidin, 1995), future studies should examine its factor structure and measurement invariance across different age groups. Next, most of our participants were mothers. This is a limitation because fathers may have different relationships with their children (Deater-Deckard and Scarr, 1996). Third, it should be noted that the internal consistency of permissive parenting was relatively low (<0.70), which is consistent with previous studies (Olivari et al., 2013, Vučković et al., 2020). Thus, caution is necessary when interpreting results related to permissive parenting. Lastly, the sample size is relatively small. Although the Hong Kong sample was composed of parents who are relatively well-off, the sample is similar to the general population in terms of household income per capita, while the SES of parents in Thailand was below the general population in terms of both monthly household income and monthly household income per capita. Future studies will need to replicate these findings in larger nationally representative samples. Despite these limitations, the results provide evidence on the structure of the abridged PSI-SF and suggest that it is a suitable instrument for measuring parental stress in Hong Kong and Bangkok.

## Conclusion

Our findings demonstrate the utility of the abridged PSI-SF for investigating parenting stress and for comparing cross-culture differences between Hong Kong and Thailand. Specifically, we find that the abridged PSI-SF exhibit a three-factorial structure in both locations. There was also evidence of good convergent and discriminant validity. We were able to establish partial metric invariance. This suggests that our parents from Hong Kong and Bangkok approached the scale in a similar way, and the scores from the three subscales can be meaningfully compared across the two locations. Notably, the findings suggest that our Hong Kong parents did not differ from their Thai counterparts in terms of parenting stress levels. This is contrary to our expectation and suggests that the ameliorating effect of traditional Thai beliefs may not be sufficient to override the pressures of parenting in contemporary Bangkok. Causes of parenting stress in different socio-cultural settings is an important issue that requires further study.

## Data Availability Statement

The datasets presented in this study can be found in online repositories. The names of the repository/repositories and accession number(s) can be found below: https://osf.io/ghtsa/?view_only=3f444b9799834cc7865f487355c3212f.

## Ethics Statement

The studies involving human participants were reviewed and approved by the Human Research Ethics Committee of the Education University of Hong Kong and the IPSR-Institutional Review Board of Mahidol University. Written informed consent to participate in this study was provided by the children’s parents or legal guardian.

## Author Contributions

XG and KL contributed to the conceptualization of the manuscript. XG was responsible for data analysis and manuscript drafting, while the corresponding author was responsible for project supervision, manuscript reviewing, and editing. Both authors contributed to the article and approved the submitted version.

## Conflict of Interest

The authors declare that the research was conducted in the absence of any commercial or financial relationships that could be construed as a potential conflict of interest.
